# Editorial: Statistical model-based computational biomechanics: applications in joints and internal organs

**DOI:** 10.3389/fbioe.2023.1232464

**Published:** 2023-06-13

**Authors:** Bhushan Borotikar, Tinashe E. M. Mutsvangwa, Shireen Y. Elhabian, Emmanuel A. Audenaert

**Affiliations:** ^1^ Symbiosis Centre for Medical Image Analysis, Symbiosis International University, Pune, India; ^2^ Department of Human Biology, Division of Biomedical Engineering, University of Cape Town, Cape Town, South Africa; ^3^ IMT Atlantique, Brest, France; ^4^ Kahlert School of Computing, University of Utah, Salt Lake City, UT, United States; ^5^ Scientific Computing and Imaging Institute, University of Utah, Salt Lake City, UT, United States; ^6^ Department of Orthopedic Surgery and Traumatology, Ghent University Hospital, Ghent, Belgium; ^7^ Department of Human Structure and Repair, Ghent University, Ghent, Belgium; ^8^ Department of Electromechanics, InViLab Research Group, University of Antwerp, Antwerp, Belgium; ^9^ Department of Trauma and Orthopedics, NHS Foundation Trust, Addenbrooke’s Hospital, Cambridge University Hospitals, Cambridge, United Kingdom

**Keywords:** computational morphometrics, osteoarthristis, foot and ankle, cerebral palsy, particle-based shape modeling, spatiotemporal modeling, automated mitral valve assessment, multi-organ statistical shape modeling

As the era of big data continues to unfold, data-driven statistical models are increasingly being applied to the study of anatomical structures, including bones, joints, and internal organs (Cootes et al., 1995; Golland et al., 2000). Statistical model-based computational biomechanics has shown immense potential in the study of joints and internal organs, shedding light on the relationships between mechanical properties, tissue structure, and function (Blanc et al., 2012). Despite their potential, these tools are not widely used in clinical applications related to joints and internal organs due to technical challenges, including handling high-dimensional data, representing nonlinear tissue behavior, addressing data heterogeneity, achieving improved generalizability for better clinical utility, and modeling anatomically valid multi-objects (joints). This Research Topic highlights state-of-the-art statistical model-based approaches used as tools in clinical applications. In this editorial, we discuss recent advances in statistical model-based computational biomechanics and their applications in studying joints and internal organs. We highlight the potential of these methods to revolutionize the diagnosis and treatment of musculoskeletal disorders and promote personalized medicine.

Bartsoen et al. proposed a workflow that combines statistical and deep learning methods to optimize implant position in Total Knee Arthroplasty (TKA) planning. This approach achieved improved results in pre-operative planning and emphasized the importance of non-invasive extraction of subject-specific ligament strains to reduce uncertainty in ligament-balanced planning. In a separate study, Van Oevelen et al. used a principal polynomial autoencoder model to nonlinearly encode cartilage wear patterns in a dataset of osteoarthritis (OA) images. They converted 3D joint space width data into 2D pixel images and compared healthy and OA cases using this approach. The study presented a novel concept of converting anatomically corresponding distance maps into pixel images for neural-based learning. The study also reconstructed a healthy virtual twin using a statistical shape model (SSM) to predict pre-morbid bone geometries of the femur and tibia bones. Using the healthy SSM-derived geometries for comparisons, the study identified four dominant orthogonal wear components (posteromedial, anteromedial, bicompartmental, and lateral) in OA that are highly correlated with relative limb geometry. Another work by Van Oevelen et al. focused on enhancing subject-specificity in computational musculoskeletal modeling by predicting various soft tissue structures around the knee, including cartilage layer, ligament anatomies, meniscal anatomy, and patellar tendon anatomy. The accuracy of these predictions was validated against manual measures, but the impact on the performance of the musculoskeletal model remains unknown.

Peterson et al. focused on developing SSMs of subtalar, talonavicular, and calcaneocuboid joints using CT scans. The study brings an interesting perspective of characterizing asymptomatic joint-level morphology and alignment differences using multi-level models, providing insights into 3D joint characteristics such as joint coverage, convergence index, and joint space distance. Another study by the same group (Khan et al.) introduced theoretical and technical problems of scalability, anatomical inconsistencies, and entangled shape statistics for multi-organ models. The study used particle-based shape modeling (PSM) and proposed a novel correspondence-point optimization of multi-organ anatomies to tackle these limitations. The study disentangles the shared space among organs, allowing for the modeling of intra-organ shape and inter-organ pose variabilities. The authors also elaborated on quantitative evaluation metrics for assessing the generality, specificity, and compactness of these multi-organ models. These two studies have improved the clinical applicability of SSMs from single-organ understanding to multi-organ understanding. A study by Cheng et al. reported differences in shape characteristics of ankle bones between healthy children and children with ankle equinus deformity due to cerebral palsy (CP). The study compared SSMs of healthy and CP cohorts and reported volumetric differences in mean shapes and subject-level differences in specific bone regions of interest. However, no precise deformation pattern could be observed for any of the three ankle bones studied.

Computational assessment of cardiovascular organs presents an increased level of technical complexity in terms of the spatiotemporal and non-linear dynamic behavior of the tissues. While large deformation models have been proposed and used in literature, they are limited to single object assessment. Lopes et al. developed an automated approach for evaluating the mitral valve apparatus in the context of transcatheter mitral valve replacement (TMVR) for mitral valve disease. They used retrospective cardiac CT scans from 50 patients to create an SSM of the left heart chambers during end-diastole. This model was then extended to other phases of the cardiac cycle to capture the dynamic changes of the mitral valve. The researchers employed anatomical landmarks to fit the model to specific cases and assess the mitral valve during end-diastolic and end-systolic phases for TMVR planning. The automated assessment was compared to manual assessment and showed an error rate of less than 5% for various mitral valve measurements. The study suggests that automated methods can improve consistency and reduce inter-observer variability, while also saving time in pre-interventional assessments. Modeling the non-linear and time-dependent shape changes of heart tissue was further refined in a study by Iyer et al. They developed a multi-organ SSM using the PSM approach to detect and extract shared boundaries between two organs. They introduced a novel technique incorporating shared boundaries within PSM for modeling multi-organ anatomies. The study included a toy dataset of parameterized shapes and a clinical dataset of biventricular heart models, specifically modeling the right ventricle, left ventricle wall, and interventricular septum. The illustrations demonstrated that the integrity of shared boundaries between multiple organs was preserved, and pathological changes could be effectively detected using this approach. Another interesting study by Adams et al. presented a spatiotemporal SSM to assess non-linear dynamic anatomies such as left atrium shape changes over the cardiac cycle. They combined an entropy-based correspondence optimization with non-linear regression—called regularized principal component polynomial regression. A 4D left atrium data was used to illustrate the model’s ability to capture underlying time dependency compared to baseline methods. The resulting SSMs have inter and intra-subject correspondence to capture a statistically significant time dependency and are agnostic to the consistency of temporal sequences across subjects.

In conclusion, data-driven statistical models have emerged as a powerful tool for evaluating anatomies, offering valuable insights into the complex relationships between morphology, function, and pathology. However, their implementation requires addressing various mathematical challenges. This special Research Topic highlights many methodological advancements in investigating the dynamic, non-linear, time-dependent, age- and gender-relevant, and subject-specific nature of joints and internal organs of the human body. While doing so, the articles published here cover a variety of topics ranging from TMVR and TKA planning, knee OA and ankle equinus due to CP, cartilage and ligament abnormalities, and cardiovascular diseases. Future research should be focused on validating the models for their clinical use, developing models that combine bone and soft tissue structures, and mechanical characterization of multi-organ deformations. Through the development of innovative mathematical approaches presented here and the refinement of existing techniques, our understanding of the human body can be expanded, leading to improved healthcare outcomes.
